# Comparative Study of Fucoidan from *Saccharina japonica* and Its Depolymerized Fragment on Adriamycin-Induced Nephrotic Syndrome in Rats

**DOI:** 10.3390/md18030137

**Published:** 2020-02-27

**Authors:** Jiaojiao Tan, Jing Wang, Lihua Geng, Yang Yue, Ning Wu, Quanbin Zhang

**Affiliations:** 1 Key Laboratory of Experimental Marine Biology, Center for Ocean Mega-Science, Institute of Oceanology, Chinese Academy of Sciences, Qingdao 266071, China; qdrdtanjiaojiao@163.com (J.T.); jingwang@qdio.ac.cn (J.W.); lhgeng@qdio.ac.cn (L.G.); yueyang@qdio.ac.cn (Y.Y.); wuning@qdio.ac.cn (N.W.); 2 Laboratory for Marine Biology and Biotechnology, Qingdao National Laboratory for Marine Science and Technology, Qingdao 266237, China; 3 University of the Chinese Academy of Sciences, Beijing 100049, China

**Keywords:** fucoidan, low-molecular-weight fucoidan, adriamycin, nephrotic syndrome

## Abstract

Nephrotic syndrome (NS) is a clinical syndrome with a variety of causes, mainly characterized by heavy proteinuria, hypoalbuminemia, and edema. At present, identification of effective and less toxic therapeutic interventions for nephrotic syndrome remains to be an important issue. In this study, we isolated fucoidan from *Saccharina japonica* and prepared its depolymerized fragment by oxidant degradation. Fucoidan and its depolymerized fragment had similar chemical constituents. Their average molecular weights were 136 and 9.5 kDa respectively. The effect of fucoidan and its depolymerized fragment on adriamycin-induced nephrotic syndrome were investigated in a rat model. The results showed that adriamycin-treated rats had heavy proteinuria and increased blood urea nitrogen (BUN), serum creatinine (SCr), total cholesterol (TC), and total triglyceride (TG) levels. Oral administration of fucoidan or low-molecular-weight fucoidan for 30 days could significantly inhibit proteinuria and decrease the elevated BUN, SCr, TG, and TC level in a dose-dependent manner. At the same dose (100 mg/kg), low-molecular-weight fucoidan had higher renoprotective activity than fucoidan. Their protective effect on nephrotic syndrome was partly related to their antioxidant activity. The results suggested that both fucoidan and its depolymerized fragment had excellent protective effect on adriamycin-induced nephrotic syndrome, and might have potential for the treatment of nephrotic syndrome.

## 1. Introduction

Chronic kidney disease has become a significant public health concern. Nephrotic syndrome (NS) is a special type of chronic kidney disease, which could be caused by a variety of factors. It is characterized by heavy proteinuria (more than 3.5 g/d), hypoalbuminemia, and edema [1]. Affected patients without effective treatment will in time develop end-stage renal disease. The adriamycin-induced nephrotic syndrome, which was first reported by Bertani et al. in 1982, is a classical nephrotic syndrome model [2]. Adriamycin is a quinone-containing anthracycline antibiotic and can be reduced to a semiquinone radical by metabolism in the kidney. The latter reacts with oxygen to produce reactive oxygen, inducing lipid peroxidation in the glomerular epithelial cells and destruction of the structure and function of the filtration membrane, and leading to progressive and irreversible proteinuria, hypoalbuminemia and hyperlipidemia [3]. An acute adriamycin-induced nephropathy model is induced by a single tail vein injection of 5–7.5 mg/kg adriamycin. This model, similar to human minimal change nephrotic syndrome, has been well characterized as an experimental model for nephrotic syndrome [4].

Treatment of nephrotic syndrome can slow its progression to end-stage renal disease. However, the therapies of nephrotic syndrome remain limited [5]. Many clinical and experimental studies have shown that the pathogenesis of nephrotic syndrome is associated with immune dysfunction. Immunosuppressive treatment, including corticosteroids, is the first-line treatment for nephrotic syndrome [6]. However, steroid resistance or steroid dependence is very common and frequently causes immune dysfunction and complicated infection, leading to end-stage renal failure [7]. Therefore identification of effective and less toxic therapeutic interventions for nephrotic syndrome remains to be an important issue.

The brown seaweed, *Saccharina japonica*, is a common seafood in China and Japan. It was documented as a traditional herb in traditional Chinese medicine for over a thousand years. The hot-water decoction of *S. japonica* is orally administered solely or combined with other herb extracts, and used for treatment of edema, a symptom of renal disease. Based on its therapeutic effect, our previous studies revealed that fucoidan, the water-soluble sulfated fucose-containing polysaccharide from *S. japonica*, was the main active component to treat edema. Fucoidan from *S. japonica* had renoprotective effect on chronic renal failure, diabetic nephropathy and acute kidney disease [8,9,10,11]. Nephrotic syndrome is a special type of chronic kidney disease, whether fucoidan have protective effect on nephrotic syndrome is still unclear.

As reviewed by Berteau and Mulloy [12], algal fucoidan represents a rather heterogeneous group of sulfated polysaccharides with complex and heterogeneous structures devoid of regularity. The structure of fucoidan extracted from *S. japonica* was much more complicated [13]. Its backbone was primarily consisted of (1→3)-linked-α-L-fucopyranose residues and a few (1→4)-α-L-fucopyranose linkages. The branch points were at C-4 of 3-linked -α-L-fucopyranose residues by β-D-galactopyranose unites or at C-2 of 3-linked -α-L-fucopyranose residues by non-reducing terminal fucose units. Sulfate groups occupied at position C-4 or C-2, sometimes C-2, 4 to fucose residues, and C-3 and/or C-4 to galactose residues. Besides fucose, fucoidan from *S. japonica* also contains minor galactose, mannose, glucose, rhamnose, and xylose. Fucoidans have been reported to have diverse bioactivities, such as antioxidant [14], anti-inflammatory [15], reno-protective [8], antitumor [16], and anticoagulant [17] activities. The molecular weight has been demonstrated to play an important role in the biological activities of polysaccharides. Comparing with unfractioned heparin, low-molecular-weight heparin has improved bio-availability, a longer half-life, and more predictable dose response, which make their use increasing common in the treatment and prophylaxis [18]. The relationship between molecular weight and bioactivities of fucoidan was reported in recent years. A low-molecular-weight fucan fraction extracted from the brown seaweed *Ascophyllum nodosum* exhibited dose-related venous antithrombotic activity [19]. A high level of inhibitory activity on complement can be achieved with low-molecular-weight fucoidan molecules [20]. If fucoidans with different molecular weight have a different effect on nephrotic syndrome still needs investigation.

In this study, we isolated a fucoidan from *S. japonica* and prepared its depolymerized fragment by oxidant degradation. The effect of fucoidan and its depolymerized fragment on adriamycin-induced nephrotic syndrome were investigated in a rat model.

## 2. Results and Discussion

### 2.1. Chemical Properties 

Fucoidan was extracted from *S. japonica* by hot-water extraction. The yield of fucoidan was 1.2%. Fucoidan was further degraded into low-molecular-weight fucoidan (LMWF) by oxidant degradation by the combination of hydrogen peroxide and ascorbic acid at room temperature. The average molecular weights of fucoidan and its depolymerized fragment LMWF were 136 and 9.5 kDa, respectively.

It is reported that ascorbate and hydrogen peroxide could induce scission of plant cell wall polysaccharides [21]. In this study, this method was used for the degradation of native fucoidan, and the changes of molecular mass indicated that fucoidan was successfully degraded into depolymerized fragment.

The chemical properties of fucoidan and its depolymerized fragment are shown in Table 1. The results indicated that both fucoidan and its depolymerized fragment had similar chemical constituents. The fucose content of fucoidan and LMWF were 31.6% and 29.6%, respectively. Their sulfate contents were 33.58% and 32.66%, respectively. Besides fucose, other monosaccharides including galactose, mannose, glucose, rhamnose, and xylose were present in fucoidan and LMWF, and they also had similar neutral monosaccharide ratios.

**Figure 1:**
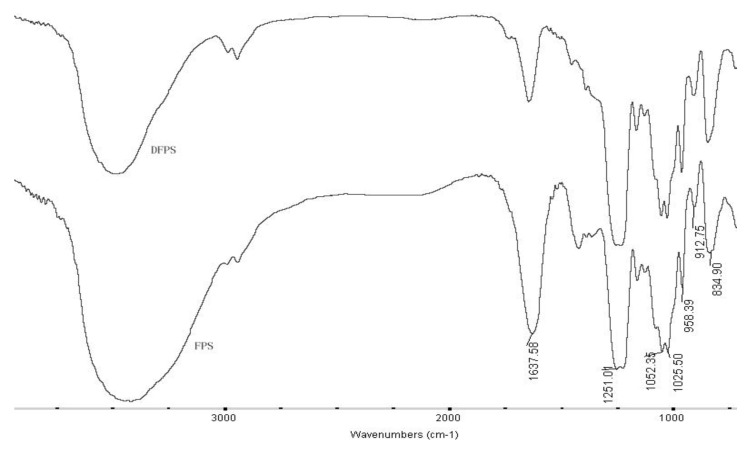
Infrared spectra of fucoidan and low molecular weight fucoidan (LMWF). FPS—fucoidan; DFPS:—low-molecular-weight fucoidan.

The IR spectra of fucoidan and LMWF are shown in Figure 1. Both samples had same infrared absorption properties, suggesting that both fucoidan and its depolymerized fragment contained the same functional groups. As shown in Figure 1, the band at 3600-3000 cm^−1^ was assigned to the deformation of O-H. The strong band around 1251 cm^−1^ was attributed to the asymmetric stretching of S=O, the absorption band around 835 cm^−1^ indicated the presence of sulfate groups. The strong absorption at approximately 1020–1050 cm^−1^ corresponded to the C-O-C/C-OH stretching frequency. Meanwhile, from the spectra, it was found that the band around 1251 cm^−1^ of fucoidan and LMWF had similar intensity, which means their sulfate contents were similar, which was consistent to the results of chemical analysis. The results indicated that oxidation degradation had no damage to the backbone structure of fucoidan.

Based on the above analysis, it can be concluded that after degradation, the chemical constituents of fucoidan and its depolymerized fragment had no significant changes, only the molecular weight was greatly decreased.

### 2.2. Evaluation of Rats Weight Alteration

The adriamycin-induced nephrotic syndrome is a classical nephrotic syndrome model. In this study, the general condition of the animals was observed during the experiments. Compared with the normal rats, rats treated with adriamycin showed abnormal behavioral activities, including reduced feed intake, easy tiredness, emaciation, tarnish, and depilation. Some animals had obvious diarrhea.

Body weight changes of all rats were examined once a week during the experiment; the weight changes before and after drug administration are shown in Table 2. Significant changes in body weight were observed among the normal and other groups during the treatment period. The body weight of the model group was significantly lower (*p* < 0.01) than that of the normal group. Compared with the model group, the body weight of rats in positive control group administrated with dexamethasone was much lower, while rats treated with fucoidan and LMWF at the dose of 100 mg/kg gained weight significantly. Rats treated with LMWF at the dose of 100 mg/kg gained a higher weight than fucoidan-treated rats at two weeks after drug administration (*p* < 0.05). LMWF treatment at the dose of 50 and 25 mg/kg also increased the body weight of rats, but had no statistical difference.

The daily feed intake of rats was significantly reduced after adriamycin treatment (Table 3). Compared with the model group, rats treated with fucoidan and LMWF at the dose of 100 mg/kg had much higher feed intake. It partly explains why these rats had higher body weight.

### 2.3. Urinary Protein Excretion

Proteinuria is the most important feature of nephrotic syndrome. To measure the urinary protein levels, the rats were placed in individual metabolic cages for 24 h urine collection once a week. We detected the 24 h urinary protein excretion of all rats. As shown in Table 4, two weeks after adriamycin administration (0 week in Table 4, the beginning time of drug administration), the 24 h urinary protein of the model group was significantly higher than that of the normal group and became higher over time. This phenomenon proved that adriamycin treatment can be used to form a rat model of nephropathy with continuous aggravation, which is similar to nephrotic syndrome. The urinary protein excretion of rats treated with fucoidan or LMWF (50 or 100 mg/kg) was significantly reduced after administration, which was significantly different from that of the model rats. The results suggested that both fucoidan and LMWF could ameliorate the symptoms of hyperalbuminuria and gradually recover the damage of glomerular filtration membrane caused by adriamycin. Compared with fucoidan, LMWF at dose of 100 mg/kg had a much lower urinary protein excretion (*p* < 0.05, three weeks after drug administration), suggesting that low-molecular-weight fucoidan could inhibit the production of proteinuria with a higher activity.

### 2.4. Blood Biochemical Indexes

At the end of the experiment, the blood biochemical indexes of rats were detected. The blood levels of total protein (TP), albumin, blood urea nitrogen (BUN), serum creatinine (SCr), total cholesterol (TC), and total triglyceride (TG) of the model rats were significantly changed (Table 5). It is worth noting that the blood lipid level of the model rats was remarkably elevated. Compared with the normal rats, the blood level of total cholesterol and total triglyceride of the model rats induced by adriamycin were increased by 9.4-fold and 6.4-fold, respectively. These results indicated that adriamycin treatment could induce significant hyperlipidemia.

Blood urea nitrogen and serum creatinine are two major indexes of renal function. As expected, adriamycin-induced rats had higher BUN and SCr levels, indicating that the kidney of the model rats was damaged by adriamycin administration. Blood total protein and albumin level of model rats were decreased by adriamycin, suggesting that hypoalbuminemia occurred in the model rats. However, the alteration of these biochemical indexes could be significantly reversed by fucoidan and LMWF treatment in a dose-dependent manner. Fucoidan, dexamethasone and LMWF (50 mg/kg) decreased the levels of BUN and SCr at the similar magnitude, while LMWF at the dose of 100 mg/kg had the highest decrease in SCr level (*p* < 0.05, vs fucoidan group). Both fucoidan and LMWF could significantly increase the blood albumin level and decrease TG and TC levels, and LMWF at the dose of 100 mg/kg had a more potent activities (*p* < 0.05). But the positive control dexamethasone could not alter the level of blood albumin, TG, and TC.

These results showed that both fucoidan and its depolymerized fragment could improve the renal function, elevate blood albumin level, and inhibit hyperlipidemia. Low-molecular-weight fucoidan had a more potent activity.

Adriamycin is a kind of amino nucleoside substance. After adriamycin transformed to semiquinone free radicals in organism, the radicals can react with oxygen to produce reactive oxygen species (ROS). The ROS further induces lipid peroxidation of glomerular epithelial cells and destroys the structure and function of the filtration membrane, leading to proteinuria and subsequent nephrotic syndrome [4]. It has been observed that a high cholesterol diet can aggravate the lipid metabolism disorder of adriamycin-induced renal injury [22]. On the contrary, serious proteinuria could lead to the incidence of hypoproteinemia, which further causes malnutrition, especially protein malnutrition. Hypoproteinemia can also reduce the plasma osmotic pressure, especially the colloidal osmotic pressure around the hepatocytes, stimulate the hepatocytes to synthesize lipoproteins, and finally results in the disorder of lipid metabolism and hyperlipidemia.

The renoprotective effect of fucoidan or LMWF may be due to its antioxidant activity. Fucoidan and low-molecular-weight fucoidan were proved to have in vitro free radical scavenging activities [23]. The antioxidant activity of fucoidan may be helpful in eliminating the reactive oxygen species initiated by adriamycin and maintaining the structure and function of glomerular basement membrane, thus inhibiting the occurrence and development of proteinuria. In order to verify the correlation between the renoprotection and anti-oxidation of fucoidan and its depolymerized fragment, we further detected the lipid peroxidation in kidney of rats.

### 2.5. Lipid Peroxidation

The levels of malondialdehyde (MDA) and superoxide dismutase (SOD) of the kidney tissue were measured in all groups. The MDA production was increased in kidney tissue of adriamycin-treated model rats (Table 6). While adriamycin treatment decreased the SOD level of the kidney tissue of rats. Treating adriamycin-induced rats with LMWF significantly and dose-dependently increased the SOD level and decreased the MDA level in kidney tissue. Fucoidan treatment also increased the SOD level and decreased the MDA level. LMWF at the dose of 100 mg/kg was more potent in decreasing the MDA level (*p* < 0.05), while both fucoidan and LMWF had similar activities on SOD at the same dose (100 mg/kg).

MDA is a main marker of endogenous lipid peroxidation [24]. The increase of MDA production indicates that peroxidative damage increases after adriamycin induction. Antioxidant enzymes are considered to be a primary defense that prevents biological macromolecules from oxidative damage. SOD is an important enzymatic antioxidant defense mechanisms that protects against oxidative processes initiated by the superoxide anion. 

The results demonstrated that both fucoidan and its depolymerized fragment were successful in inhibiting lipid peroxidation in the kidney of adriamycin-induced rats, as observed in the reduction of MDA production and increase of SOD level. The results were in accordance with previous studies on the antioxidant activities of fucoidan and low-molecular-weight fucoidans [24]. The renoprotective effect of fucoidans was at least partly due to its antioxidant activities.

### 2.6. Effect of Molecular Weight on Adriamycin-Induced Nephrotic Syndrome

The molecular weight has been demonstrated to play an important role in the biological activities of polysaccharides [25]. Low-molecular-weight heparin is a classic example. Comparing with unfractioned heparin, low-molecular-weight heparin has improved bio-availability, a longer half-life, and more predictable dose response, which make its use increasingly common in the treatment and prophylaxis of thromboembolism [18]. High and low-molecular-weight fucoidans were also reported to have differential effect on the severity of collagen-induced arthritis in mice [26]. A daily oral administration of high-molecular-weight fucoidan (HMWF, 100 ± 4 kDa) enhanced the severity of arthritis, inflammatory responses in the joint cartilage, and the levels of collagen-specific antibodies, while low-molecular-weight fucoidan (LMWF, 1 ± 0.2 kDa) reduced the severity of arthritis and the levels of Th1-dependent collagen-specific IgG2a. 

Our group previously reported that fucoidan from *S. japonica* has a protective effect on chronic renal failure, diabetic nephropathy, and acute kidney injury [8,9,10,11]. This study revealed that fucoidan and low-molecular-weight fucoidan also have a protective effect on adriamycin-induced nephrotic syndrome. LMWF at the same dose had a better protective effect than fucoidan. Since fucoidan and LMWF had similar sugar constituents but different molecular weight, the difference in renoprotection between fucoidan and LMWF was mainly attributed to their discrepancy in molecular weight. The exact mechanism of the renoprotective effect of fucoidan and LMWF needs further investigation in the following study.

## 3. Materials and Methods

### 3.1. Materials

Adriamycin was purchased from Meilun Biological Technology Co., Ltd. (Dalian, China). Dexamethasone Acetate tablet (Lot No.170545) was purchased from Tianjin Tianyao Pharmaceutical Co. Ltd. (Tianjin, China). All other chemicals and reagents were obtained from general commercial sources.

### 3.2. Preparation of Fucoidan and Its Depolymerized Fragment

*Saccharina japonica* Aresch (Formerly name: *Laminaria japonica*, Laminariaceae), cultured in the coast of Rongcheng, Shandong Province, China, was collected in July 2017. The fresh seaweed was washed with clean seawater and sun dried. Fucoidan was extracted from *S. japonica* in hot water at 100 °C for 2 h. The extraction liquid was filtered and 1% CaCl_2_ was added to precipitate soluble alginate. After centrifugation, the supernatant was dialyzed using a dialysis membrane (molecular weight cutoff (MWCO) 3500 Da) against pure water, concentrated under reduced pressure and finally precipitated with ethanol. The precipitate was washed twice with ethanol and dried to get fucoidan.

Low-molecular-weight fucoidan (LMWF) was prepared using free radical degradation with the combination of hydrogen peroxide and ascorbic acid. Briefly, fucoidan was dissolved in distilled water to a final concentration of 0.5%, ascorbic acid and hydrogen peroxide was added to the solution to a final concentration of 30 mM, and stirred at room temperature for 2 h. The solution was filtered, dialyzed against pure water (MWCO 3500 Da), concentrated under reduced pressure and subsequently precipitated with ethanol (75%, final concentration).

### 3.3. Chemical Analysis

Fucose content was determined using cysteine hydrochloride–sulfuric acid method with L-fucose as the standard [27]. Sulfate content was analyzed using ion chromatography with potassium sulfate as the standard. Total sugar content was measured by phenol-sulfuric acid method. Uronic acid was tested using modified carbazole method with D-glucuronic acid as the standard [28]. Monosaccharide composition was estimated with high-performance liquid chromatography (HPLC) according to the method of Zhang et al. [29]. The proportion of monosaccharide was evaluated by calculating the molar ratio of other monosaccharide to fucose. Infrared spectra were recorded with a Nicolet-750 FT-IR spectrometer. Average molecular weight was determined by high performance gel filtration chromatography (HPGPC) on TSK G4000 PW_xl_ or G3000 PWxl column eluted with 0.7% Na_2_SO_4_ using a series of dextrans with different molecule weight as standard.

### 3.4. Animals and Experimental Design

Adult male Sprague-Dawley rats (220–240 g) were provided by Experimental Animal Center of Shandong University. The animals were maintained on a 12 h dark/light cycle at about 22 °C and relative humidity (60–70%), allowed free access to standard rat chows and water during the experiments. The experiments were performed in complete compliance with the National Guide for the Care and Use of Laboratory Animals, and were approved by the Experimental Animal Ethics Committee of Institute of Oceanology, Chinese Academy of Sciences, China (approval code SCXK(Lu)20190002).

The adriamycin-induced nephrotic syndrome model in rats was performed according to Bertani et al. protocol [2]. A single dose of adriamycin (6.0 mg/kg body weight) was injected via the femoral vein to induce nephrotic syndrome (NS) model. After injection of adriamycin, 24 h urinary protein content was measured once a week. Two weeks following the adriamycin injection, proteinuria was detected, then the rats were randomly divided into six groups (*n* = 10): Group 1 was the model group which consisted of NS rats; Group 2 consisted of 10 NS rats and the rats were treated with daily oral gavage of fucoidan at dosage of 100 mg/kg body weight; Group 3, 4, and 5 consisted of NS rats and were treated daily oral gavage of LMWF at dosage of 100, 50, and 25 mg/kg body weight, respectively. Group 6 was the positive control group, and NS rats in this group were administrated with dexamethasone acetate at dose of 0.1 mg/kg body weight. Group 7 was the normal group (*n* = 10) and rats were orally administrated with 10 mL/kg/d of saline. All rats were raised in the same environment and were allowed unlimited access to water and conventional rat chow during the experiments. During the experiments, the animals were weighed twice a week, the general state of animals was recorded every day, including body hair, stool, and mental state.

To measure urinary protein levels, the rats were placed in individual metabolic cages for 24 h urine collection once a week.

On the thirtieth experimental day the animals were anaesthetized by ether and blood samples were taken from the eye-pit of rats. After the blood sample was taken, the rats were killed and the kidneys were weighted and taken for routine histological examination.

### 3.5. Blood Biochemical Indexes

Serum was separated and the levels of serum creatinine, blood urea nitrogen (BUN), total protein, albumin, total cholesterol, and total triglyceride were analyzed with Beckman biochemical analyzer. The protein of the kidney was determined using Coomassie Brilliant Blue (G-250) by the method of Bradford using a bovine serum albumin (BSA) standard.

### 3.6. Assessment of MDA and SOD Levels

The kidney levels of SOD and MDA were analyzed using kits from NanJing JianCheng (NanJing JianCheng Bio Inst, China), and the protocols were all followed the introduction of the kit.

### 3.7. Statistical Analysis

Data were presented as means ± SD. ANOVA was used to analyze the data and the Student’s t-test was used to determine the level of significance of differences in population means. A significant difference was accepted with *p* < 0.05.

## 4. Conclusions

Fucoidan was extracted from *S. japonica*, and further degraded into low-molecular-weight fucoidan by oxidant degradation. The average molecular weights of fucoidan and low-molecular-weight fucoidan were 136 and 9.5 kDa, respectively. The in vivo animal experiment revealed that fucoidan and low-molecular-weight fucoidan had protective effect on adriamycin-induced nephrotic syndrome. Fucoidan and LMWF treatment could significantly inhibit adriamycin-induced proteinuria and decrease the elevated BUN, SCr, TG, and TC level of rats in a dose-dependent manner. LMWF at the same dose had a better protective effect than fucoidan. Since fucoidan and LMWF had similar sugar constituents but different molecular weight, the difference in renoprotection between fucoidan and LMWF was mainly attributed to their discrepancy in molecular weight. Their renoprotective effect maybe partly related to their antioxidant effect.

## Figures and Tables

**Table 1:** Chemical constituents of fucoidan and low-molecular-weight fucoidan (LMWF) prepared from *S. japonica.*

Samples	Fucose %	Uronic Acid %	Sulfate %	Neutral Monosaccharide (Molar Ratio)					
				Fuc	Gal	Man	Glc	Rha	Xyl
LMWF	31.60	5.69	33.58	1.000	0.303	0.088	0.072	0.035	0.053
Fucoidan	29.12	6.07	32.66	1.000	0.296	0.068	0.087	0.039	0.046

**Table 2:** Effect of fucoidan and low molecular weight fucoidan (LMWF) treatment on body weight of rats ($\bar{X}$ ± *S*, g).

Groups	Dosage (mg/kg)	Weeks after Drug Administration				
		0	1	2	3	4
Normal	-	344.4 ± 12.2	366.3 ± 21.9	387.5 ± 23.5	401.2 ± 24.8	408.8 ± 26.9
Model	-	296.2 ± 26.9 ^ΔΔ^	293.0 ± 16.7 ^ΔΔ^	294.4 ± 19.8 ^ΔΔ^	303.9 ± 19.6 ^ΔΔ^	309.5 ± 21.8 ^ΔΔ^
dexamethasone	0.1	293.2 ± 30.8	250.0 ± 18.6 ^**^	223.3 ± 14.1 ^**^	225.9 ± 16.3 ^**^	225.4 ± 14.2 ^**^
Fucoidan	100	292.8 ± 15.3	315.5 ± 24.4 *	307.7 ± 14.1	323.3 ± 11.3 ^*^	332.9 ± 17.1 ^*^
LMWF	100	279.8 ± 13.0	318.6 ± 29.4 ^*^	329.3 ± 31.5 ^*^	332.9 ± 34.1 ^*^	339.5 ± 35.9 ^*^
LMWF	50	286.7 ± 21.3	309.6 ± 29.7	321.5 ± 30.5 ^*^	315.1 ± 33.2	317.3 ± 37.9
LMWF	25	285.2 ± 19.3	309.5 ± 20.2	320.6 ± 24.8 ^*^	319.9 ± 27.8	314.5 ± 28.1

^△^: *p* < 0.05, ^△△^: *p* < 0.01 (vs normal group); *: *p* < 0.05, **: *p* < 0.01 (vs model group).

**Table 3:** Effect of fucoidan and low molecular weight fucoidan (LMWF) treatment on feed intake of rats ($\bar{X}$ ± *S*, g).

Groups	Dosage (mg/kg)	Weeks after Drug Administration				
		0	1	2	3	4
Normal	-	26.8 ± 1.3	36.1 ± 1.0	34.0 ± 0.9	37.2 ± 0.3	38.1 ± 1.3
Model	-	16.8 ± 0.3 ^Δ^	23.1 ± 0.1 ^Δ^	23.9 ± 1.4 ^Δ^	24.0 ± 0.9 ^Δ^	23.7 ± 0.4 ^Δ^
dexamethasone	0.1	16.9 ± 1.6 ^Δ^	17.4 ± 0.0 ^*^	19.4 ± 3.4	16.6 ± 1.9	19.1 ± 2.9
Fucoidan	100	17.4 ± 1.4 ^Δ^	28.2 ± 2.0 ^*^	29.5 ± 2.1 ^*^	32.0 ± 2.0 ^*^	32.7 ± 3.8 ^*^
LMWF	100	18.3 ± 1.6 ^Δ^	32.9 ± 2.7 ^*^	30.5 ± 1.0 ^*^	31.9 ± 5.0 ^*^	34.9 ± 2.4 ^*^
LMWF	50	18.5 ± 0.7 ^Δ^	23.5 ± 1.6	29.3 ± 1.3	28.0 ± 5.4	28.4 ± 0.9
LMWF	25	18.1 ± 1.8 ^Δ^	22.9 ± 2.4	28.2 ± 0.9	26.7 ± 4.9	24.7 ± 1.8

^△^: *p* < 0.05 (vs normal group); *: *p* < 0.05 (vs model group).

**Table 4:** Effect of fucoidan and LMWF treatment on 24 h urinary protein of rats ($\bar{X}$ ± *S*, mg/mL).

Groups	Dosage (mg/kg)	Weeks after Drug Administration (mg/mL)				
		0	1	2	3	4
Normal	-	0.58 ± 0.40	0.73 ± 0.51	0.73 ± 0.47	0.65 ± 0.35	0.59 ± 0.36
Model	-	22.42 ± 22.55 ^ΔΔ^	36.60 ± 14.54 ^ΔΔ^	43.98 ± 26.64 ^ΔΔ^	48.41 ± 22.18 ^ΔΔ^	54.60 ± 31.27 ^ΔΔ^
dexamethasone	0.1	19.98 ± 14.87 ^ΔΔ^	23.56 ± 10.47 *	25.25 ± 9.85 *	26.63 ± 16.98 *	28.62 ± 19.15 *
Fucoidan	100	20.45 ± 10.00 ^ΔΔ^	20.19 ± 12.37 *	23.21 ± 13.54 *	26.13 ± 9.63 *	27.82 ± 12.28 *
LMWF	100	20.11 ± 10.86 ^ΔΔ^	19.26 ± 12.12 *	21.88 ± 13.28 *	21.71 ± 12.76 ^**^	25.31 ± 9.58 ^*^
LMWF	50	23.28 ± 19.18 ^ΔΔ^	22.90 ± 12.39 *	24.40 ± 7.89 *	29.34 ± 9.67 *	29.15 ± 8.79 *
LMWF	25	25.16 ± 20.65 ^ΔΔ^	33.19 ± 15.96	30.42 ± 21.03	31.00 ± 14.17	36.91 ± 20.51

^△^: *p* < 0.05, ^△△^: *p* < 0.01 (vs normal group); *: *p* < 0.05, **: *p* < 0.01 (vs model group).

**Table 5:** Effect of fucoidan and low molecular weight fucoidan (LMWF) on blood biochemical indexes of NS rats ($\bar{X}$ ± *S*).

Groups	Dosage (mg/kg)	T-P (g/L)	ALB (g/L)	SCr (µmol/L)	BUN (mmol/L)	TG (mmol/L)	T-CHO (mmol/L)
Normal	-	68.63 ± 5.77	32.71 ± 8.78	77.23 ± 5.26	7.21 ± 0.92	0.48 ± 0.13	1.50 ± 0.38
Model	-	50.85 ± 18.50 ^Δ^	16.14 ± 3.68 ^ΔΔ^	101.33 ± 20.96 ^ΔΔ^	16.14 ± 7.60 ^ΔΔ^	4.99 ± 1.38 ^ΔΔ^	11.12 ± 3.07 ^ΔΔ^
dexamethasone	0.1	59.34 ± 16.39	18.87 ± 4.95	86.37 ± 3.18	10.41 ± 2.42 ^*^	3.80 ± 2.02	9.48 ± 3.20
Fucoidan	100	64.46 ± 6.94	21.40 ± 6.61 *	85.60 ± 11.42	10.52 ± 1.59 ^*^	2.86 ± 1.29 ^**^	8.10 ± 4.01
LMWF	100	66.49 ± 11.28 ^*^	26.26 ± 10.11 *	79.53 ± 15.50 ^*^	8.64 ± 2.93 ^*^	2.39 ± 1.54 ^**^	7.23 ± 2.99 ^**^
LMWF	50	61.86 ± 4.16	22.50 ± 8.27 *	86.77 ± 10.29	10.64 ± 2.28	2.90 ± 1.38 ^**^	8.01 ± 1.71 ^**^
LMWF	25	61.35 ± 4.53	19.93 ± 2.85 *	79.85 ± 4.88	9.40 ± 1.55 ^*^	2.94 ± 1.73 ^**^	8.63 ± 2.84 ^*^

^△^: *p* < 0.05, ^△△^: *p* < 0.01 (vs normal group); *: *p* < 0.05, **: *p* < 0.01 (vs model group). T-P: total protein; ALB: albumin; SCr: serum creatinine; BUN: blood urea nitrogen; TG: triglyceride; T-CHO: total cholesterol.

**Table 6:** Effect of fucoidan and low molecular weight fucoidan (LMWF) on malondialdehyde (MDA) and superoxide dismutase (SOD) levels in kidney ($\bar{X}$ ± *S*).

Groups	Dosage (mg/kg)	MDA (nmol/mg prot)	SOD (u/mg prot)
Normal	-	1.03 ± 0.35	86.31 ± 5.50
Model	-	1.65 ± 0.66 ^Δ^	72.92 ± 12.66 ^Δ^
dexamethasone	0.1	1.07 ± 0.35 *	77.48 ± 11.22
Fucoidan	100	1.12 ± 0.44 *	84.45 ± 10.44 *
LMWF	100	0.94 ± 0.33 **	83.72 ± 8.15 *
LMWF	50	1.16 ± 0.27 *	82.89 ± 7.27 *
LMWF	25	1.37 ± 0.22	79.68 ± 9.91

^△^: *p* < 0.05 (vs normal group); *: *p* < 0.05, **: *p* < 0.01 (vs model group).
